# Intussusception among Japanese children: an epidemiologic study using an administrative database

**DOI:** 10.1186/1471-2431-12-36

**Published:** 2012-03-22

**Authors:** Masato Takeuchi, Toshio Osamura, Hideo Yasunaga, Hiromasa Horiguchi, Hideki Hashimoto, Shinya Matsuda

**Affiliations:** 1 Department of Pediatrics, The University of Tokyo Hospital, Tokyo, Japan; 2 Department of Pediatrics, Kyoto Second Red Cross Hospital, Kyoto, Japan; 3 Department of Health Management and Policy, Graduate School of Medicine, The University of Tokyo, Tokyo, Japan; 4 Department of Health Economics and Epidemiology Research, School of Public Health, The University of Tokyo, Tokyo, Japan; 5 Department of Preventive Medicine and Community Health, University of Occupational and Environmental Health, Fukuoka, Japan

## Abstract

**Background:**

The epidemiology of intussusception, including its incidence, can vary between different countries. The aim of this study was to describe the epidemiology of childhood intussusception in Japan using data from a nationwide inpatient database.

**Methods:**

We screened the database for eligible cases ≤ 18 years of age, who were coded with a discharge diagnosis of intussusception (International Classification of Diseases, 10th revision: K-561) between July to December in 2007 and 2008. We then selected cases according to Level 1 of the diagnostic certainty criteria developed by the Brighton Collaboration Intussusception Working Group. We examined the demographics, management, and outcomes of cases, and estimated the incidence of intussusception.

**Results:**

We identified 2,427 cases of intussusception. There were an estimated 2,000 cases of infantile intussusception annually in Japan, an incidence of 180-190 cases per 100,000 infants. The median age at diagnosis was 17 months, and two-thirds of the patients were male. Treatment with an enema was successful in 93.0% of cases (2255/2427). The remainder required surgery. Secondary cases accounted for 3.1% (76/2427). Median length of hospital stay was 3 days. Of the 2,427 cases, we found 2 fatal cases associated with intussusception.

**Conclusions:**

This is currently the largest survey of childhood intussusception in Asia using a standardized case definition. Our results provide an estimate of the baseline risk of intussusception in Japan, and it is higher than the risk observed in other countries.

## Background

Intussusception is the most common cause of intestinal obstruction among infants and young children, and can also affect older children and adolescents [1]. Improvements have been made in the diagnosis and treatment of intussusception [2]; however, poor outcomes can still occur, even in developed countries [3].

In 1999, Rotashield (the first-generation rotavirus vaccine licensed in the United States) was withdrawn from the market because of a potential increased risk of intussusception [4]. Two recent studies investigated whether the second-generation Rotavirus vaccine was also associated with an increased risk of intussusception [5,6], and revealed conflicting results. One post-marketing survey reported a small but statistically significant increased risk [5], while the other study found no evidence of an elevated risk of intussusception [6]. The potential risk of intussusception among vaccinated children has motivated researchers to estimate the baseline risk of intussusception [7,8]. In the last several years, a growing number of studies have been published focusing on the incidence of intussusception [9-11]. Several surveys have also been published regarding the incidence of intussusception in Eastern Asia including Taiwan, Hong Kong and Vietnam [12]. However, the results vary, ranging from 70 to 300 cases per 100,000 children, suggesting that there are regional differences in the incidence of intussusception.

The primary goal of our study was to describe the epidemiology of childhood intussusception in Japan, including patients' demographic characteristics, management, and outcomes, and to estimate the baseline incidence of infantile intussusception before the introduction of the Rotavaccine program.

## Methods

### Data source

Our survey was based on data from the Diagnosis Procedure Combination (DPC) inpatient database in Japan. The DPC database is a nationwide database of inpatients that contains administrative claims data and discharge abstracts. Data are collected for 6-month periods (between July 1 and December 31) each year. For the analyses, we used data obtained in 2007 and 2008. In 2008, for example, data were compiled from approximately 2.9 million inpatients at 855 hospitals. This represented 45% of hospitalized acute care cases in Japan.

The database includes data on diagnoses, comorbidities at admission and complications after admission coded using *International Classification of Diseases, 10th Revision *(ICD-10) codes, surgical procedures, length of stay, discharge status including in-hospital death, and costs [13,14].

Because this study was based on a secondary analysis of the anonymous patient database, the requirement for informed consent was not applicable. Study approval was obtained from the institutional review board of the University of Occupational and Environmental Health.

### Criteria

All children aged between 0 and 18 years old admitted with intussusception (ICD-10 code: K561) were screened. At this point, patients with various diagnostic conditions were included. We used criteria developed by the Brighton Collaboration Intussusception Working Group to categorize patients into 5 groups (definitive to not a case), using a mix of major and minor criteria [8]. Although these criteria were primarily developed for vaccine safety data, they are also highly reliable for epidemiological research (sensitivity: 97%; specificity: 87-91%) [15]. Because our database did not contain information about clinical signs and symptoms, we included only cases meeting the clinical case definition of definite (level I of diagnostic certainty) intussusception according to surgical, radiological, or autopsy criteria [8], and excluded all other cases coded as intussusception in the database. A previous study ensured that this approach enabled us to capture more than 90% of cases with intussusception correctly [10].

Readmissions to the same hospital shortly after discharge (e.g., within a week) may involve cases with either insufficient reduction or with true recurrence. In this regard, Daneman et al. reported that most of the recurrence occurred within a few days following enema reduction [16]. Accordingly, we considered all admissions for intussusception to the same hospital as independent events.

### Estimation of population-based incidence of infantile intussusception

We estimated the population-based incidence of intussusception among infants who are the target population for Rotavirus vaccination (Table 1). The coverage rate of the DPC database was 45% and it was uncertain whether patient distribution was balanced between DPC hospitals and non-DPC hospitals; therefore, sampling bias due to the referral pattern may be present in our dataset. In this regard, we assumed that patients were equally distributed if the hospital volumes were similar, regardless of hospital type. Based on this assumption, we classified DPC hospitals into four categories according to their hospital bed volumes; similarly, hospitals throughout Japan were categorized in this manner. We then calculated the estimated number of intussusception cases each year (Y*i*) and 95% confidence intervals (CI) by applying the following equation using Wald CIs for the population proportion:

**Table 1:** Estimation of Population-based Incidence of Infantile Intussusception

Hospital Volume	No. of hospitals in Japan	No. of beds in Japan (Ni)	No. of beds in DPC participating hospitals (ni)		No. of intussusception from July to December (Xi)		Estimated No. of cases per year (Yi)	
			**2007**	**2008**	**2007**	**2008**	**2007**	**2008**

< 400 beds (i = 1)	7,001	566,658	138,979	119,853	86	97	701 (597-806)	917 (788-1046)

400 - < 600 beds (i = 2)	466	175,715	98,050	89,627	176	169	631 (565-697)	663 (592-733)

600 - < 800 beds (i = 3)	161	88,870	54,351	49,740	98	78	320 (276-365)	278 (235-322)

> 800 beds (i = 4)	95	78,995	50,245	50,245	97	71	305 (262-348)	223 (187-260)

Total	7,723	910,238	341,625	309,465	457	415	1,957 (1743-2216)	2,081 (1802-2361)

$${\text{Y}}_{i}/{\text{N}}_{i}=\text{ p}\pm \text{1}.\text{96}\surd \text{p}\left(\text{1}-\text{p}\right)/{\text{n}}_{i}$$

where *i *(1 to 4) is the number of each strata, N*_i _*is the number of beds in all acute care hospitals in Japan, n*_i _*denotes the number of beds in the DPC hospitals and p = 2X*_i_*/n*_i _*(X*_i _*is the observed number of intussusception cases in DPC hospitals between July and December each year). In this estimation, we assumed that there was no seasonality in the incidence of intussusception [17] and doubled the half-year incidence (X*_i_*) to obtain the whole-year incidence.

In 2007 and 2008, the number of births was 1,090,000 each year. Therefore, we used this number as the denominator of the population-based incidence of infantile intussusception.

### Statistical analysis

Descriptive statistics were used to summarize the demographic characteristics of patients. To describe the characteristics of the patients, means (± standard deviation) or medians (± interquartile range) are reported where appropriate. Fisher's exact test was used to calculate 95% CI.

All statistical analyses were performed using SPSS for Windows 17.0 (SPSS, Chicago, IL, USA) and R 2.10.0 (available at http://www.r-project.org).

## Results

### Characteristics of cases

A total of 2,427 cases with intussusception were identified; 1,185 in 2007 and 1,242 in 2008. During the same time period, the number of all-cause admissions of patients ≤ 18 years of age was 626,770. Thus, intussusception accounted for 0.39% of all-cause admissions (Table 2). The annual number of cases of intussusception among Japanese infants was estimated as 1,957 (95%CI: 1743-2216) in 2007 and 2081 (95%CI: 1802-2361) in 2008 (Table 1). This corresponded to an incidence of 179 (95%CI: 165-203) per 100,000 infants in 2007 and 191 (95%CI: 165-216) in 2008.

**Table 2:** All-cause admissions, all intussusceptions and secondary intussusceptions in each age group

Age (y)	All-cause admissions	Intussusceptions (per 1,000 admissions)	Secondary cases
0	157,494	872 (5.5)	14(1.6%)

1	88,125	657 (7.5)	8 (1.2%)

2	51,900	415 (8.0)	12 (2.9%)

3-4	74,689	300 (4.0)	7 (2.3%)

5-12	157,099	148 (0.9)	22 (14.9%)

13-18	97,463	35 (0.4)	13 (37.1%)

Total	626,770	2,427 (3.9)	76 (3.1%)

**Figure 1:**
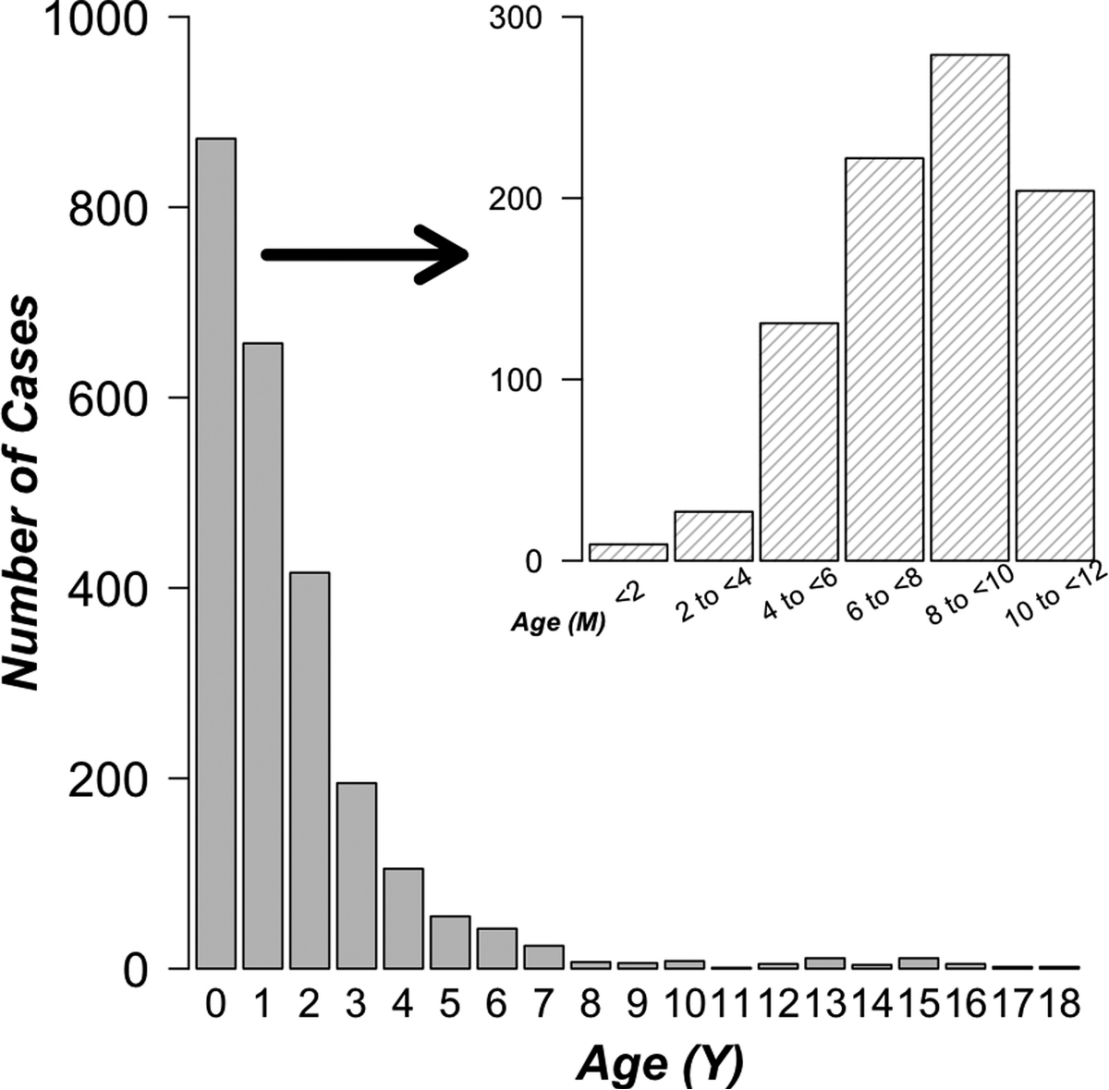
**Age distribution of all intussusception cases (N = 2,427)**. Inset, Age distribution of intussusception cases in infants (0 to < 12 months; n = 872).

Of the 2,427 patients, 1,610 (66.3%) were male. The median age at the time of diagnosis was 17 months (interquartile range: 9.5-31.5) and 92.5% (2244/2427) of cases were under 5 years of age (Figure 1). Intussusceptions occurred most frequently in the first year of life (35.9%: 872/2427) with a peak incidence between 8 and 10 months of age, but were rarely found under 3 months of age (*n *= 9, including 3 neonatal cases).

Among the 2,427 cases with intussusceptions, 93.0% (2,255) were successfully reduced with an enema, and the remaining 175 cases required surgical management. In the surgical intervention group, bowel resection was required in 52 cases (29.7% of the surgical cases). The median length of hospital stay was 3 days (median: 3 days in the non-surgical group and 8 days in the surgical group).

We found that 36 patients (37 episodes) had been readmitted to the same hospital within a week after treatment of the initial intussusception (mean: 2.3 days). At the second admission, 32 cases of intussusception were successfully reduced by enema and 5 required surgery. In the surgical cases, underlying conditions were found in 2 children with polyps; none were reported to have perforation.

### Secondary causes of intussusception

We found 76 (3.1%) intussusceptions due to secondary causes (Table 3). The remaining patients were thought to be idiopathic cases. Frequencies of secondary cases were 1-3% under 5 years of age, and increased thereafter. Schönlein-Henoch purpura, Meckel's diverticulum and polyps were the three major causes of the pathological lead points (PLPs) of these 76 cases. Intussusception associated with Schönlein-Henoch purpura mainly affected young children, while Meckel's diverticulum and polyps were present in children of all age groups.

**Table 3:** Pathological Lead Point (N = 77)

Causes	Number of Patients	Age (yrs)
**Structural**		

Meckel's diverticulum	13	0-13

Duplication cyst	2	4-10

Malrotation	3	0-1

Hirschsprung disease	1	0

Mesenteric hernia	1	6

**Vascular/hematological**		

Schönlein-Henoch purpura	15	1-7

Hemolytic-uremic syndrome	4	2-3

Idiopathic thrombocytopenic purpura	1	0

Lymphoma	2	15-16

Nephrotic syndrome	3	2-7

Leukemia	1	12

Kawasaki Disease	1	1

**Neoplasms**		

Polyps	8	0-15

Benign tumor	3	6-13

Malignant tumor	3	0-15

Tumor (details not available)	3	0-18

**Others**		

Appendicitis/Appendix	7	1-13

Postoperative	2	0

Foreign Body	1	1

Congenital biliary dilation + Pancreatitis	1	5

Endometriosis	1	15

### Complications

We identified 27 (1.1%) patients with complications related to intussusception. These complications included perforation and/or peritonitis (n = 9), systemic infections such as sepsis and meningitis (n = 8), shock (n = 5), seizures (n = 3) and death (n = 2). Intussusception-associated complications occurred irrespective of patient age.

We found 2 fatal cases attributed to intussusception; thus, the mortality rate in our series was 0.08% (2/2427, 95%CI: 0.01-0.30%). The first patient was a 3-year-old girl. She was in cardiopulmonary arrest on arriving at hospital and died within 24 hours after presentation. Autopsy revealed invagination of the intestine. The second was a 2-year-old boy. His invagination was reduced successfully with a non-surgical procedure, but he died of complicated hemolytic-uremic syndrome on the second hospital day. Autopsy was not performed in the second case.

## Discussion

Our study elucidates the nature of childhood intussusception in Japan. To our knowledge, this is the first study to investigate the incidence of intussusception among an Asian population using a nationwide database combined with the Brighton Criteria for diagnostic accuracy.

Recent analyses of childhood intussusception in Western countries [1,18,19] found that the peak age was between 4 and 9 months; the male:female ratio was around 2:1; PLPs were found in 2.6-15% of cases; and non-surgical enema reductions were successful in 80-95% of patients. Our findings are essentially consistent with these previous reports.

We found that intussusceptions accounted for 3.9 cases per 1,000 all-cause admissions (8.0 cases in 2-year-olds and 0.12 in 18-year-olds). This hospital-based incidence appears to be higher than those observed in Europe (0.66-2.24 per 1,000 children in inpatient departments) [7]. Similarly, we estimated that the population-based incidence of intussusception was 179-191 per 100,000 infants in our cohort. Only one study is currently available regarding the population-based incidence of infantile intussusception in Japan [20]. This earlier study showed that the incidence was 185 cases per 100,000 infants based on a 25-year survey conducted in one small region of Japan. This result is in accordance with our estimation and gives further support to our findings. Our estimated incidence was much higher than those in other countries (179-191 vs 30-80 cases per 100,000 population) [12]. Because both the hospital and population-based incidence rates were higher than those of other countries, it is very likely that the incidence of intussusception in Japan is high compared with other countries. Our results also suggest that the incidence of intussusception cannot be easily extrapolated to other countries, because studies from Eastern Asian countries outside Japan reported that the incidence of intussusception was 70-300 cases per 100,000 children [12]. With respect to the incidence of intussusception, surveys in each region may be essential.

Our study has several advantages. First, we used a standardized case definition of intussusception [8]. This enabled us to compare epidemiologic surveys from other areas and temporal trends in the same area. Reported incidence rates of intussusceptions vary among different populations and times [1,21,22]. Thus, standardized case definitions can contribute to maximizing the reliability of epidemiologic data. Second, our study, including over 2,000 patients, is large enough to determine the epidemiology of intussusception. Third, our study determined the case-fatality rate. Little information is available about the mortality rate in developed countries [23]. The mortality rate in our study was 0.08% (95%CI: 0.01-0.30%), which is comparable to data available from the United States reporting 18-56 deaths per 100,000 cases [3]. Furthermore, our data included epidemiologic data of older children and adolescents, which is lacking in previous studies. This study thus provides a deeper insight into intussusception in children of all ages. In addition, our survey included rare but severe complications such as perforation/peritonitis, systemic infections (including sepsis, bacterial meningitis), and neurological involvement. Although previous case reports have sporadically reported these complications [23-25], this study estimates their incidence rates based on cross-sectional data.

Several limitations of the current study should be acknowledged. First, because only inpatient data was included, we may have failed to capture cases treated in emergency units or outpatient clinics. Outpatient management of children with intussusception is widely practiced in some countries [5,26]. In Japan, however, in-hospital observation is recognized as the standard practice even after successful reduction. Thus, it seems unlikely that we failed to capture a number of cases with intussusception. Second, there is inherent limitation in observational studies using administrative databases. For example, some clinical information was not included in our database (e.g., patients' past and present history, site of intussusception, or types of contrast media used). Another aspect of this limitation is that our study may be susceptible to systemic bias; referral bias, for example, may be present in our dataset. Therefore, our results should be interpreted in the context of the limitations arising from the nature of this study. Finally, our database collected data only over 6 months because of the cost to participating hospitals. Therefore, we could not investigate whether there were seasonal trends of intussusception in Japan. This limitation could influence the estimation of the population-based incidence of intussusception. A prospective patient registration survey may be needed using a standardized case definition such as the criteria set out by the Brighton Collaboration.

## Conclusions

This study describes the epidemiology of childhood intussusception in Japan, including current information on incidence data, pathological lead points and complications. We also highlight that the observed incidence of intussusception among Japanese infants is higher than those reported from other countries. Until active surveillance of intussusception is available, the nationwide administrative database has a potential role in monitoring the incidence of childhood intussusception.

## Competing interests

The authors declare that they have no competing interests.

## Authors' contributions

MT performed data analysis and manuscript preparation. TO was involved in the design of this work. HY, HH (Dr. Horiguchi), HH (Dr. Hashimoto) and SM were responsible for providing the database. All authors read and approved the final draft.

## Pre-publication history

The pre-publication history for this paper can be accessed here:

http://www.biomedcentral.com/1471-2431/12/36/prepub
